# The fish ability to accelerate and suddenly turn in fast maneuvers

**DOI:** 10.1038/s41598-022-08923-5

**Published:** 2022-03-23

**Authors:** Damiano Paniccia, Giorgio Graziani, Claudio Lugni, Renzo Piva

**Affiliations:** 1 Department of Mechanical and Aerospace Engineering, University of Rome ”La Sapienza”, Rome, Italy; 2 CNR-INM, Marine Technology Research Institute, Rome, Italy; 3 Institute of Marine Hydrodynamics, Harbin Engineering University, Harbin, China; 4 NTNU-AMOS, Center for Autonomous Marine Operation Systems, Trondheim, Norway

**Keywords:** Biomechanics, Fluid dynamics, Biomimetics

## Abstract

Velocity burst and quick turning are performed by fish during fast maneuvers which might be essential to their survival along pray–predator encounters. The parameters to evaluate these truly unsteady motions are totally different from the ones for cruising gaits since a very large acceleration, up to several times the gravity, and an extreme turning capability, in less than one body length, are now the primary requests. Such impressive performances, still poorly understood, are not common to other living beings and are clearly related to the interaction with the aquatic environment. Hence, we focus our attention on the water set in motion by the body, giving rise to the relevant added mass and the associated phenomena in transient conditions, which may unveil the secret of the great maneuverability observed in nature. Many previous studies were almost exclusively concentrated on the vortical wake, whose account, certainly dominant at steady state, is not sufficient to explain the entangled transient phenomena. A simple two-dimensional impulse model with concentrated vorticity is used for the self-propulsion of a deformable body in an unbounded fluid domain, to single out the potential and the vortical impulses and to highlight their interplay induced by recoil motions.

## Introduction

The aquatic motion of fish is characterized by paths of long term cruising swimming and by very fast maneuvers during pray–predator encounters, either for escaping or for foraging needs. Such maneuvers, for instance the so called C-start characterized by a C shape bending, give rise to a sudden change of the swimming direction together with a huge acceleration leading the fish to follow a proper path to survive or to capture the desired pray^1^. Their purpose is very different from the one for standard cruising and the usual performance parameter, i.e. the cost of transport given by the ratio between expended power and locomotion speed^2,3^, is no longer a priority so that different measures are needed to search for optimal performances. The most common fast start swimming gaits were largely described in a survey paper by Domenici and Blake^4^ with a large set of experimental data, very useful for understanding the relevant phenomena. In particular, both C-start and S-start maneuvers are deeply analyzed, but we will concentrate here only on the first one since, in our opinion, it is more rich of interesting aspects like the sudden change of the swimming direction. Anyhow, a full comprehension of many facets of the physical behavior is still not available and a satisfactory account of all the reasons for such unique achievements is still missing. Some recent and very interesting contributions^5–7^ investigated a problem, in a way related to the present one, concerning the peculiar acceleration properties of the octopus that is propelled by a water jet expelled by the body itself, which in the mean time experiences a simultaneous reduction of its volume. The reported results for this case show a dominant effect of the added mass reduction which acts as a substantial improvement of the propulsion due to the water jet. Along with the proper differences essentially due to the unavoidable recoil motions^8–10^, the C-start under investigation may be brought back to the above problem since the added mass and its variability may play a central role for the maneuver’s performance. For the analysis, we have to consider the full system of the evolution equations for the kinetic variables pertaining to the body center of mass. Since the numerical results may be quite involved, we consider a simple impulse model with concentrated vorticity, so to isolate the potential and the vortical contributions as a *conditio sine qua non* for a proper physical interpretation of the results. As a major goal, we intend to confirm the added mass and its variability to be key items during the transient phase though the release of vorticity is always significant to definitely prevail at the end of the maneuver. Other interesting contributions analyzed the C-shape deformation accompanied by a traveling wave from head to tail to show, by numerical results, a more impressive performance^11–14^. Also in this case, we intend to provide the proper reasons for the increased efficiency of the maneuver. In the following sections we report and discuss a few numerical results for a deeper understanding of fast start swimming maneuvers whose comprehension, beyond a basic value per se, may provide a technical contribution to the design of biomimetic fishlike robots for particular applications requiring excellent maneuverability.

## Results

**Figure 1:**
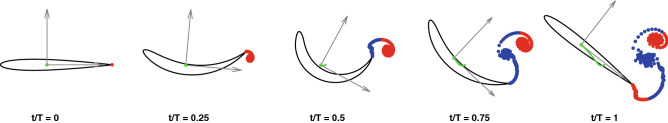
Snapshots of the C-start maneuver of a neutrally buoyant fish from the numerical simulation. The relative animation is reported in Movie S1.

As a first step, let us make a short description of the C like fast start just to recall by a few snapshots (see Fig. 1 and the related animation reported in Movie S1) the main phases of this pretty elaborated maneuver which obeys to the conservation of both linear and angular momenta, since no external actions are applied. The fish willing to suddenly accelerate and change its swimming direction initiates a preparatory phase via a rotation of its tail which induces a simultaneous opposite rotation of the body fixed frame.

**Figure 2:**
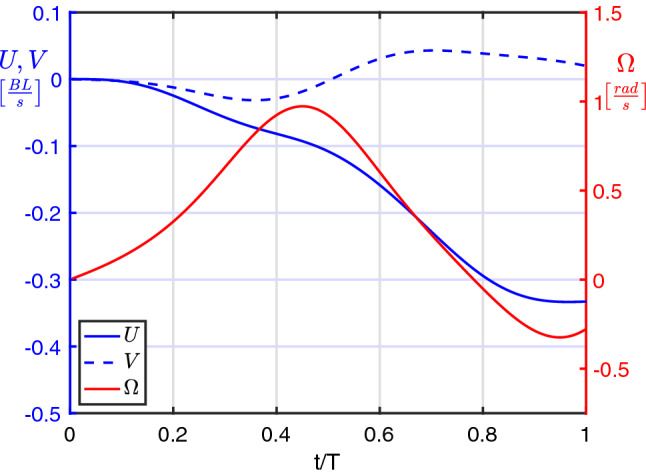
Velocity components for the C-start maneuver of a neutrally buoyant fish.

The successive propulsive phase, corresponding to the rapid return of the tail to the position aligned with the forward axis, gives rise to a substantial velocity boost in the same direction while the whole motion is accompanied by a significant release of vorticity. The kinematic performance of the C-start maneuver for a neutrally buoyant fish may be furtherly appreciated by the velocity components reported in Fig. 2 where we see that during the preparatory phase, i.e. for $0 \le t/T < 0.5$ when the tail is raised towards the head (see Fig. 1), the body fixed frame starts to counter-rotate with an angular velocity $\Omega$ whose maximum occurs approximately for $t/T = 0.5$. A relatively small forward velocity *U* from right to left (i.e. negative in sign) is also obtained halfway, but a much larger forward speed is finally achieved at the end of the propulsive phase when the tail is pushed back. No comments are made about the lateral velocity component *V* since, in a first approximation, its presence is quite negligible.

**Figure 3:**
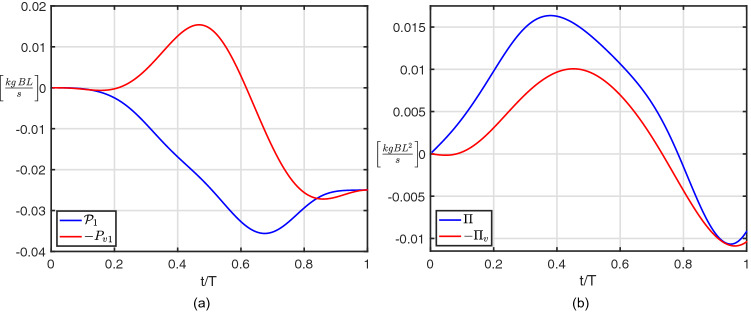
Fluid impulses for C-start maneuver: (**a**) total forward impulse ${{\mathcal {P}}}_1$ and its vortical contribution ${P_v}_1$; (**b**) total angular impulse ${\Pi }$ and its vortical contribution ${\Pi _v}$.

The literature on the subject was mostly focused on the study of the vortex shedding and of the vortical wake geometry as a potential source of comprehension, while a little attention was given to the added mass that we consider instead of primary importance for the maneuver. For a quantitative evaluation of all these contributions, we rely on the conservation of the linear impulse along the forward direction:

1 $$\begin{aligned} (m + m_{11})\,U = {{\mathcal {P}}}_1 \end{aligned}$$

where *m* is the body mass and $m_{11}$ is the added mass coefficient as properly defined when deriving the full system of equations (12) reported in the Methods section. Namely, Eq. (1) represents the first equation of the system once all the contributions but the one containing the unknown forward velocity *U* are grouped together within a single term ${{\mathcal {P}}}_1 = - {P_v}_1 - {P_{sh}}_1 - m_{12} V - m_{13} \Omega$ to ease the interpretation of the results. Specifically ${{\mathcal {P}}}_1$, beyond the component ${P_v}_1$ associated to the shed vortices and ${P_{sh}}_1$ associated to the shape deformation, includes the coupling terms given by the lateral and angular velocities times the proper added mass coefficients $m_{12}$ and $m_{13}$, respectively. We may easily appreciate from Fig. 3a the very large difference between ${{\mathcal {P}}}_1$ and its vortical component, obviously covered by the left aside terms whose large impact on the maneuver clearly appears. It is interesting to evaluate the relative weight of the various terms to highlight the overwhelming predominance of the coupling term $-m_{13}\Omega$ which provides a substantial momentum transfer from the angular to the forward direction (diagrams reported in Fig. S2a). All the terms covering the above difference are shown to become negligible at the end of the propulsive phase where the deformation is over and the fish returns to its straight configuration. Actually, in this condition the total and vortical impulses ${{\mathcal {P}}}_1$ and ${-P_v}_1$ perfectly coincide, hence we may assess that the value of the final swimming velocity at the end of the C-start maneuver may be obtained by accounting only for the shed vortices contribution^15^. At the same time, the vortical wake is shown to be unable to give a correct picture of the global physical phenomenon since all the other terms, in a way related to the added mass, have a dominant influence during the transient phase. By following the same reasoning, let us write the equation for the angular momentum:

2 $$\begin{aligned} (I_{zz} + m_{33}) \Omega = {\Pi } \end{aligned}$$

where, as before, the term ${\Pi }$ is grouping together all the other contributions but the one containing the angular velocity $\Omega$, while $I_{zz}$ is the body moment of inertia and $m_{33}$ is the proper added mass coefficient. Analogously, the difference between ${\Pi }$ and its vortical contribution ${-\Pi _v}$, reported in Fig. 3b, shows again the relevance of the left aside terms on the maneuver with a special regard to the coupling ones due to added mass (reported in Fig. S2c). At this point, since we have verified the limited role of the vortical wake for understanding the C-start, we may now pass to the dynamics of the maneuver to account for the effects of the added mass variability. Namely, by taking the time derivative of Eq. ((1)), we obtain

3 $$\begin{aligned} \frac{dU}{dt} = \frac{1}{m + m_{11}}\frac{d{{\mathcal {P}}}_1}{dt} -\frac{U}{m + m_{11}}\frac{dm_{11}}{dt} \end{aligned}$$

where the acceleration $\frac{dU}{dt}$ is split into two forcing terms. The first one depends directly on the time derivative of the forward impulse ${{\mathcal {P}}}_1$, while the second one depends on the time derivative of the added mass coefficient $m_{11}$ along the forward direction. Both terms on the right hand side of Eq. ((3)) are divided by the sum of the body mass and of its added mass coefficient $m_{11}$. Hence, the added mass coefficient accounting for all the water set in motion by the body forward translation behaves like the body mass, i.e. the smaller its value, the more effective are the forcing terms on the body acceleration. Moreover, the time derivative of the added mass coefficient $m_{11}$ appears also as a forcing term which, for a reducing value of $m_{11}$, may provide a boost in the body forward velocity, as highlighted by Spagnolie and Shelley^16^.

**Figure 4:**
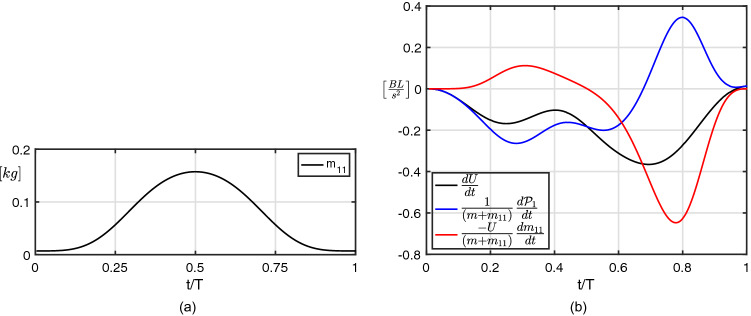
Time history of (**a**) the added mass coefficient $m_{11}$ and of (**b**) the forward acceleration contributions for the C-start maneuver.

**Figure 5:**
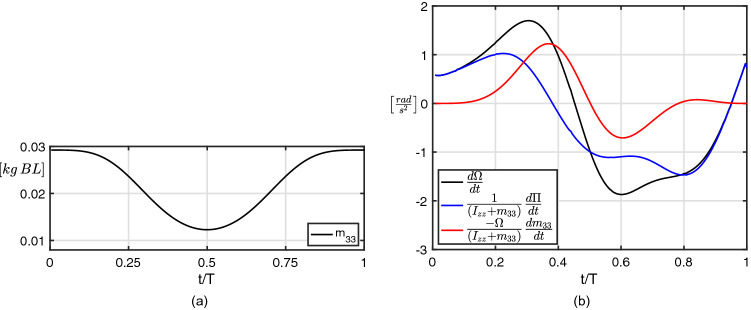
Time history of (**a**) the added mass coefficient $m_{33}$ and of (**b**) the angular acceleration contributions for the C-start maneuver.

By proceeding in an analogous way, similar equations may be obtained for the lateral and angular velocity components but, since the lateral velocity is much smaller and less important than the angular one, we report here only the expression for the angular acceleration:

4 $$\begin{aligned} \frac{d\Omega }{dt} = \frac{1}{I_{zz} + m_{33}}\frac{d\Pi }{dt} -\frac{\Omega }{I_{zz} + m_{33}}\frac{dm_{33}}{dt} \end{aligned}$$

where the first term on the right hand side depends on the time derivative of the angular impulse $\Pi$, while the second one accounts for the variation of the added mass coefficient $m_{33}$. For an easier understanding of the effects due to the added mass variation on the forward and the angular acceleration experienced by the fish, we reported in Figs. 4a and 5a, respectively, the time history of the added mass coefficients $m_{11}$ and $m_{33}$ while the behaviour of all the other coefficients is reported for completeness in Fig. S3. The total forward and angular accelerations and their contributions as given by Eqs. (3) and (4) are reported in Figs. 4b and 5b. In the first one, i.e. Fig. 4b, we observe how the two combined contributions always give rise to an acceleration from right to left (with a negative sign in our frame of reference) until the end of the maneuver. To this regard, according also to Fig. 4a, the time history of the second term on the r.h.s. of Eq. ((3)), accounting for the added mass variation, represents the main source of acceleration in the forward direction along the propulsive phase, even though a lighter opposite acceleration, substantially a drag, is shown during the preparatory phase. At the same time, the term accounting for $\frac{d{{\mathcal {P}}}_1}{dt}$ shows a quite similar but opposite behaviour since the favorable effect appears during the preparatory phase, while the resistive effect occurs during the propulsive phase. By looking at the different components of $\frac{d{{\mathcal {P}}}_1}{dt}$ reported in the Supplementary Material (see Fig. S4a), we have a further assessment of the dominant role played by the coupling among the angular and the forward velocities. With regard to the angular acceleration reported in Fig. 5b, the term related to the variation of the added mass coefficient $m_{33}$ goes along with the time derivative of the angular impulse $d\Pi /dt$ for most of the entire maneuver. The cooperative action of these two terms enhances the fish capability to perform quick turnings leading to a large angular velocity $\Omega$ which also has a favourable influence on the forward velocity through the coupling terms included in ${{\mathcal {P}}}_1$ as reported for completeness in Fig. S4b.

**Figure 6:**
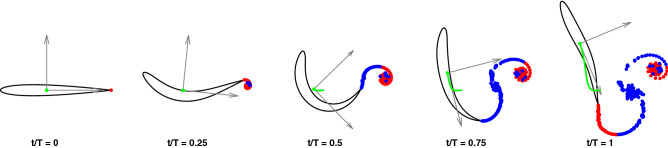
Snapshots of the C-start maneuver combined with a wave undulation from the numerical simulation. The relative animation is reported in the Movie S2.

**Figure 7:**
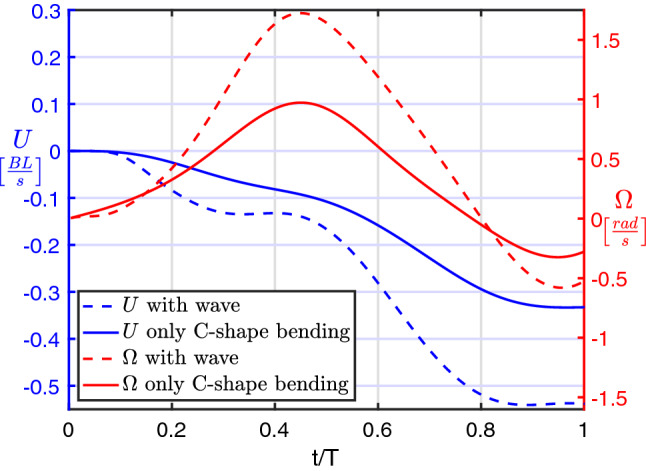
Comparison between forward and angular velocity components for the C-start maneuver with and without wave undulation.

The presence of an undulatory motion cooperating with the main C-shape bending fully maintains the validity of the above reasoning about the relevance of the added mass for a good maneuverability. Indeed, the addition of a proper traveling wave is even enhancing the full deformation by leading, on the one side, to larger values of the added mass coefficients together with their time variation and, on the other side, to an increase of the angular velocity, which keeps providing the predominant forward momentum transfer. The increased deformation involving a larger amount of water to be accelerated was also mentioned by Gazzola et al.^17^ as a fostering effect for the C-start performance. The snapshots in Fig. 6 and the related animation in Movie S2 give a first glance evaluation of the more efficient maneuver, while the diagrams in Fig. 7 show the larger forward and angular velocities compared with the ones without traveling wave. Further figures on this case, quite similar to the previous ones for the basic C-start, are reported in Figs. S5 and S6.

## Discussion

The surprising performances that fish may reach when engaged in fast start maneuvers have attracted the attention of biologists, physicists and engineers since such events are not fully understood and, even more, they are far from being reproduced by the actual more advanced technologies. It was our intention to analyze, as the most significant sample case, the C-start of a fishlike body by a simple impulse model which is instrumental to evaluate separately the various contributions for the accomplishment of the maneuver. Several previous studies^14,15,17–20^ analyzed in detail position and strength of the shed vortices with the intent to draw some hints for a sound physical interpretation of the fish maneuvering performance. For instance, Epps and Techet^15^ investigated the vortical wake released during the C-start and measure the complete variation of the fish linear momentum through the linear momentum of the released vortices. In other words, by evaluating the momentum associated to the vortex clusters right after the fast start, they manage to estimate the swimming velocity at the end of the maneuver. However, as suggested by many authors^21–24^ and clearly stated by Zhang^25^, the shed vortices behave like terrestrial footprints hence they are not telling the whole story about the fish dynamics. The debate on this point is quite subtle and we like to add a further deepening by reporting the main findings obtained by our numerical simulations. On the one hand, the vortical contributions, even though eventually dominant, are shown to be unable to explain all the intermediate steps of the maneuver. On the other hand, the added mass and its variation are proven to have, in aquatic environment, the larger impact on the extreme accelerations and on the high turning capabilities. Apart from assessing the key role of the reducing value of the coefficients $m_{11}$ and $m_{33}$, we did show the prevailing action of the mutual momentum transfer between the angular and the forward direction due to the coupling terms related to the mixed coefficients. Among these terms which involve the proper recoil motions, the one associated to the angular velocity is shown to have the largest influence on the entire maneuver. No qualitative changes were observed when the C-shape bending was accompanied by a traveling wave along the fishlike body as usually observed in nature and repeatedly reported in the literature^11–14^. From a quantitative point of view, it has to be mentioned that, when a traveling wave is prescribed, the maneuver performances are even more impressive. From the above numerical results and from their analysis, we are able to draw a quite straight conclusion. In a nutshell: when considering truly unsteady motions like the fast start of a deformable body in aquatic environment, the vortical wake is not sufficient to catch the essence of the maneuver as it would be in presence of a light fluid like air, but the multiple effects associated to the added mass, though vanishing at the end of the maneuver, are prevailing for the description of the transient phase and for the realization of the maneuver itself.

## Materials and methods

We study the motion of a fish ${\mathcal {B}}$ swimming in a quiescent fluid within an unbounded fluid domain $\mathcal {V}$. The self propelled motion is generated by the internal forces and moments exchanged between the swimming body and the surrounding fluid. These actions are expressed through the impulse formulation to avoid the convergence problems actually appearing for the momentum in unbounded fluid domains^26,27^. We consider the planar, two-dimensional motion of an impermeable, flexible body (with density $\rho _b$) whose bounding surface $S_b$ is moving with velocity $\varvec{u}_b$ in an incompressible flow field with density $\rho$ and velocity $\varvec{u}$ vanishing at the far field boundary. By using well known vector identities for the unbounded two-dimensional fluid volume^28–31^, the linear fluid impulse is defined as

5 $$\begin{aligned} \mathbf {p} \,\,= \int _{\mathcal {V}} \rho \, \varvec{u} \, dV = \frac{1}{N-1} \left[ \int _{\mathcal {V}}{\varvec{x}} \times \varvec{\omega } \, dV + \int _{S_b}{\varvec{x}}\times (\varvec{n}\times \varvec{u}^+) \, dS \right] \end{aligned}$$

where *N* is the dimension (here $N = 2$ is assumed) and $\varvec{x}$ is the position vector in the inertial frame. In Eq. (5), $\varvec{\omega }$ is the vorticity, $\varvec{u}^+$ stays for the limiting value of the fluid velocity on $S_b$ and the integral over the external boundary receding to infinity has been proven to exactly vanish (Wu^28^, Wu et al.^31^, Noca et al.^32^). The normal $\varvec{n}$ points out of the flow domain ${\mathcal {V}}$ which encloses all the vorticity. The right-hand side of Eq. (5) is independent of the choice of the reference frame origin^30–32^.

Similarly, the angular impulse is

6 $$\begin{aligned} \begin{aligned} \mathbf {\pi } \,\,= \int _{\mathcal {V}} \rho \, \varvec{x} \times \varvec{u} \, dV = \frac{1}{2} \left[ \int _{V} {|x|}^2 \varvec{\omega }\, dV + \int _{S_b} {|x|}^2 (\varvec{n} \times \varvec{u}^+ ) \, dS \right] \end{aligned} \end{aligned}$$

We consider here the moment with respect to a given pole, so $\varvec{x}$ is the generic distance of the field point from the pole. Due to the absence of external forces the total linear and angular momenta are conserved and, by assuming null initial conditions, we have

7 $$\begin{aligned} \int _{\mathcal {B}} \rho _b \, \varvec{u}_b \, dV + \mathbf {p} = 0 \end{aligned}$$

8 $$\begin{aligned} \int _{\mathcal {B}} \rho _b \, \varvec{x} \times \varvec{u}_b \, dV + \mathbf {\pi } = 0 \end{aligned}$$

where the forces acting on the body and on the fluid are obtained by time differentiating the two terms appearing in (7), respectively. The motion of the body can be expressed as the sum of the prescribed shape deformation with velocity $\varvec{u}_{sh}$ plus the translational ($\varvec{u}_{cm}$) and rotational ($\varvec{\Omega }$) velocity of the frame with origin in the centre-of-mass.

9 $$\begin{aligned} \varvec{u}_b = \varvec{u}_{sh} + \varvec{u}_{cm} + \varvec{\Omega } \times \varvec{x^\prime } \end{aligned}$$

where $\varvec{x^\prime }$ is the position vector in the body frame, i.e.: $\varvec{x} = \varvec{x}_{cm} + \varvec{x^\prime }$. The prescribed deformation of the body has to conserve linear and angular momenta, as formally given by $\int _{\mathcal {B}} \rho _b \varvec{u}_{sh} \, dV = 0$ and $\int _{\mathcal {B}} \rho _b \varvec{x}^\prime \times \varvec{u}_{sh} \, dV = 0$. By combining the expression of $\varvec{u}_b$ with Eqs. (7) and (8) we obtain

10 $$\begin{aligned} m \, \varvec{u}_{cm} + \, \mathbf {p} = 0 \end{aligned}$$

11 $$\begin{aligned} I_{zz} \, \Omega + {\pi } = 0 \end{aligned}$$

where *m* and $I_{zz}$ are the inertial properties of the body. We may then express $\varvec{p}$ and ${\pi }$, via a Helmholtz decomposition, in terms of their potential and vortical contributions as $\mathbf {p} = \mathbf {p}_\phi + \mathbf {p}_v$ and ${\pi } = {\pi }_\phi + {\pi }_v$, where the added mass effects are embedded within the potential impulses $\mathbf {p}_\phi$ and ${\pi }_\phi$ while the vortical impulses $\mathbf {p}_v$ and ${\pi }_v$ are related to the vortex sheet around the body and to the vortices shed into the wake^33–35^. A complete and detailed description of the procedure can be found in Paniccia et al.^36^, where all the steps up to the final system of equations written in the body fixed frame are reported to obtain the two linear velocity components *U* and *V* and the angular velocity $\Omega$

12 $$\begin{aligned} \left\{ \begin{aligned} \left( m_{11} + {m} \right) \,U + m_{12} V + m_{13} \Omega&= -{{P}_{sh}}_1 - {P_{v}}_1 \\ m_{21} \,U + \left( m_{22} + {m} \right) V + m_{23} \Omega&= -{{P}_{sh}}_2 - {P_{v}}_2 \\ m_{31} \,U + m_{32} V + \left( m_{33} + {I_{zz}} \right) \Omega&= -\Pi _{sh} - \Pi _{v} \end{aligned} \right. \end{aligned}$$

where the added mass coefficients $m_{ij}$, which are usually fully embedded into the forcing terms for standard CFD simulations^37^, are here easily obtained by the following definition

13 $$\begin{aligned} m_{ij} = - \int _{S_b} \phi _j\frac{d\phi _i}{dn}\,dS \end{aligned}$$

In the above system the potential impulses have been split into some terms related to the unknown rigid body motions, which are expressed through the added mass coefficients, and other terms with the subscript *sh*, due to the shape deformation, which remain on the r.h.s. of the equations together with the vortical contribution. The flow solutions are obtained by an unsteady potential code for a slender body^38^ while vortex shedding from the trailing edge is taken into account by a classical unsteady Kutta condition^39^. This well-known numerical procedure has been extensively used in the literature to study rigid bodies like airfoils moving with a fully prescribed motion while we study here the free swimming of a deformable body^40,41^ which presents a much larger complexity since the linear and angular rigid body velocities are now unknown. A short description of the prescribed deformation is reported in the Supplementary Material together with the specific data for the numerical simulation collected in Table S1.

## Supplementary Information

Supplementary Information 1.

Supplementary Information 2.

Supplementary Information 3.

## Data Availability

All data generated or analysed during this study are included in this published article (and its supplementary information files).
