# Supplementary material for: The fish ability to accelerate and suddenly turn in fast maneuvers
Source: Sci Rep. 2022 Mar 23;12:4946. doi: 10.1038/s41598-022-08923-5 (PMC8943085; doi:10.1038/s41598-022-08923-5)
Supplement: Supplementary file 1 — Supplementary Information 1. [file 41598_2022_8923_MOESM1_ESM.pdf]

SUPPLEMENTARY MATERIAL - The fish ability to accelerate and suddenly turn in fast maneuvers

Damiano Paniccia[1,*,+], **Giorgio Graziani**[1,+], **Claudio Lugni**[2,3,4,+], **and Renzo Piva**[1,+]

[1]Dept. of Mechanical and Aerospace Engineering, University of Rome ”La Sapienza”, Rome, Italy
[2]CNR-INM, Marine Technology Research Institute, Rome, Italy
[3]Institute of Marine Hydrodynamics, Harbin Engineering University, Harbin, China
[4]NTNU-AMOS, Center for Autonomous Marine Operation Systems, Trondheim, Norway
[*]damiano.paniccia@uniroma1.it
[+]these authors contributed equally to this work

Deformation and data for the numerical simulations

The C-start maneuver is mainly characterized by a C-shaped oscillatory bending through which the fish accelerates the surrounding fluid to obtain a large increase of the forward velocity and an impressive turning capability. The C bending deformation is usually accompanied by an undulatory deformation which is eventually leading to a more efficient maneuver[1–4]. In the present work we focused on the basic phenomena underlying the outstanding fish performance by means of a mathematical and a numerical model able to isolate and separate all the different physical contributions. To this purpose, we used the impulse formulation to satisfy the conservation of the total linear and angular momenta for a self-propelled deformable body and we obtained the numerical solutions by means of a two-dimensional panel method with concentrated vortex shedding through an unsteady Kutta condition. Both the mathematical and numerical models have been deeply described in Paniccia et al.[5].
The fish body is represented by a NACA0010 airfoil while its prescribed deformation is taken from Liu et al.[4] and briefly described in the following. The deformation is given by the curvature $k(s,t)$ of the airfoil midline and, in general, it is divided in two parts

$$k(s,t) = k_b(s,t) + k_w(s,t) \tag{S1}$$

where $k_b(s,t)$ is the part associated to the main C-shaped oscillatory bending and $k_w(s,t)$ is a curvature traveling wave. Specifically, the main bending is defined as

$$k_b(s,t) = \begin{cases} 0 & 0 \le s < 0.1 \\ a_b\left(1 - \cos\left(2\pi \frac{t}{T}\right)\right) & s \ge 0.1 \end{cases} \tag{S2}$$

where a_b is the maximum bending amplitude and T is the time period of the entire maneuver. The curvature traveling wave is given as

$$k_w(s,t) = a_w A(s)\tau(t)\sin\left(2\pi\left(s - \frac{t}{T}\right) + \psi\right) \tag{S3}$$

where a_w is maximum wave amplitude and the ramp function $\tau(t)$ is assigned as

$$\tau(t) = \begin{cases} \frac{t}{T_r} - \frac{1}{2\pi}\sin\left(\frac{2\pi t}{T_r}\right) & 0 \le t \le T_r \\ 1 & t \ge T_r \end{cases} \tag{S4}$$

where T_r is the duration of the ramp and $A(s)$ is an amplitude modulation is assigned as

$$A(s) = \begin{cases} 0 & 0 \le s < 0.1 \\ s^2 - 0.2s + 0.5 & s \ge 0.1 \end{cases} \tag{S5}$$

The values of all the different parameters used for the numerical simulations are collected in Table S1. The corresponding deformations in the body-fixed frame are reported in fig.S1 for the preparatory and the propulsive phase. To be notice the significant difference among the two phases once the undulatory deformation is considered.

Table S1. Parameters for numerical simulations

	bending only	bending + traveling wave
a_b	0.465π	0.465π
T	2	2
a_w	0	1.4
ψ	0	π
T_r	0	0.4

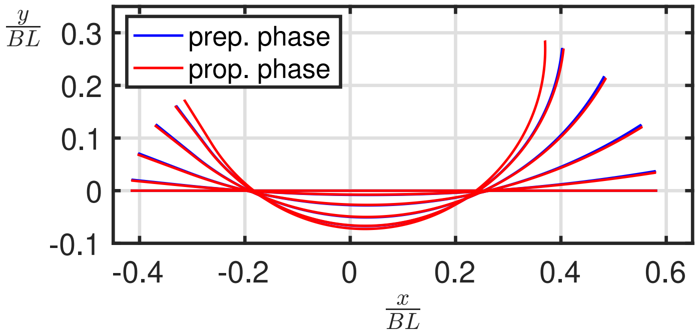

(a)

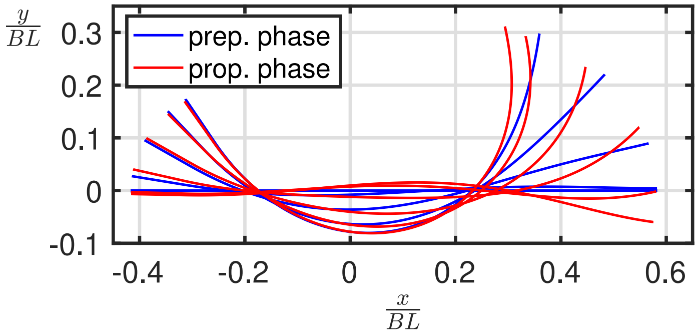

(b)

Figure S1. Midline envelope of the C-start deformation: (a) bending only and (b) bending plus traveling wave.

Additional Results

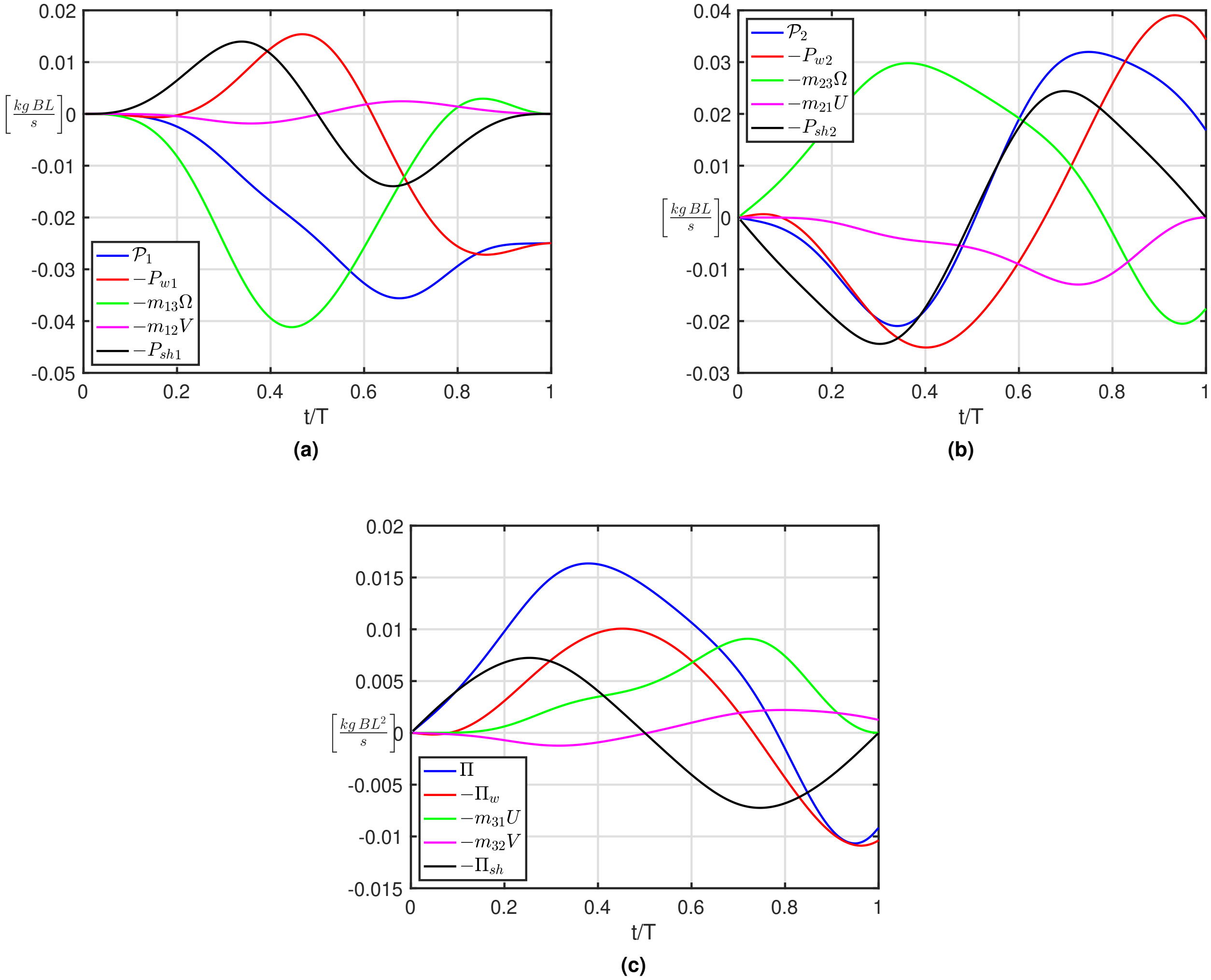

Figure S2. Time history of the impulses contributions for the C-start maneuver: (a) forward, (b) lateral and (c) angular direction. The blue and red curves in fig.S2a and fig.S2c, appearing also in fig.3a and fig.3b, are here compared with all the other impulse contributions not in the main text. It is worth to notice the large impact of the green curve in fig.S2a representing the momentum transfer from the angular to the forward direction discussed in the main text. With regard to the lateral impulses reported in fig.S2b, they are not mentioned in the main text due to the relatively small importance of the lateral velocity for the maneuver.

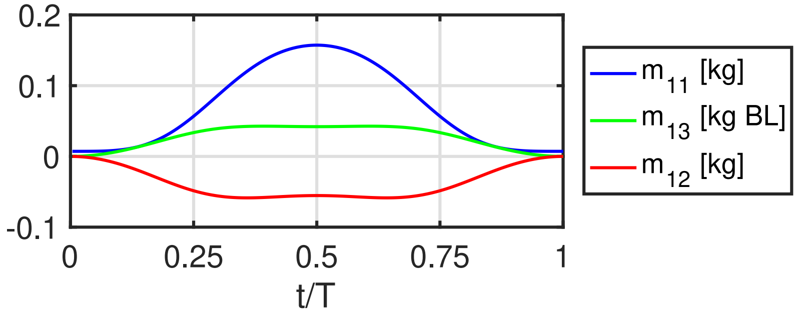

(a)

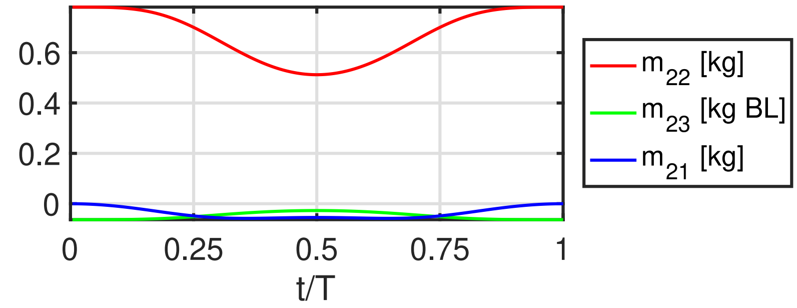

(b)

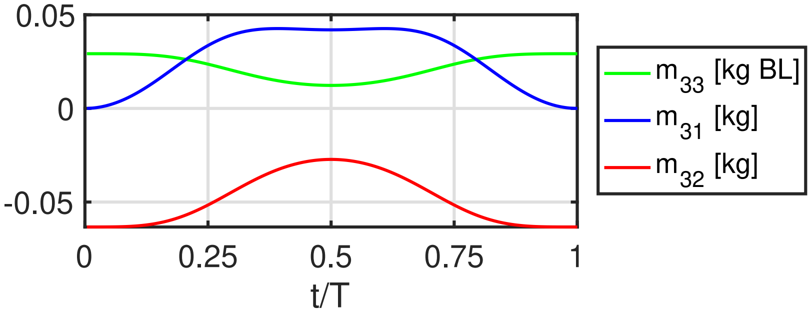

(c)

Figure S3. Time history of the added mass coefficients m_{ij} for the C-start maneuver governed by eq.(12) in the main text: (a) added mass coefficients m_{1j} appearing in the equation along the forward direction, (b) added mass coefficients m_{2j} appearing in the equation along the lateral direction and (c) added mass coefficients m_{3j} appearing in the equation along the angular direction. Notice the very large added mass coefficient m_{22} leading to a very small lateral velocity as reported in the main text.

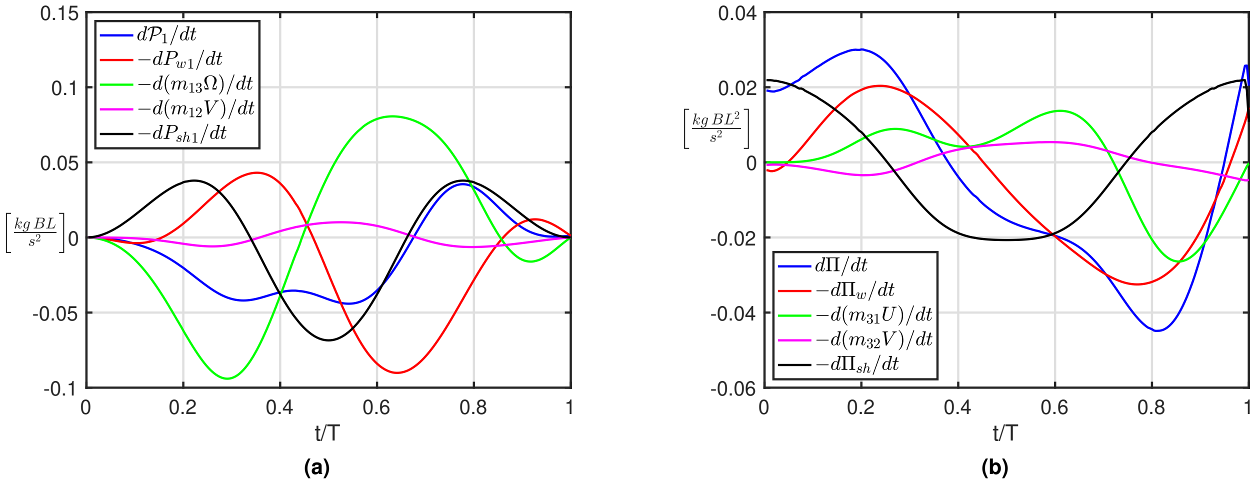

Figure S4. Splitting of the time derivative of (a) the total forward impulse term $\mathscr{P}_1$ and (b) the total angular impulse term Π.

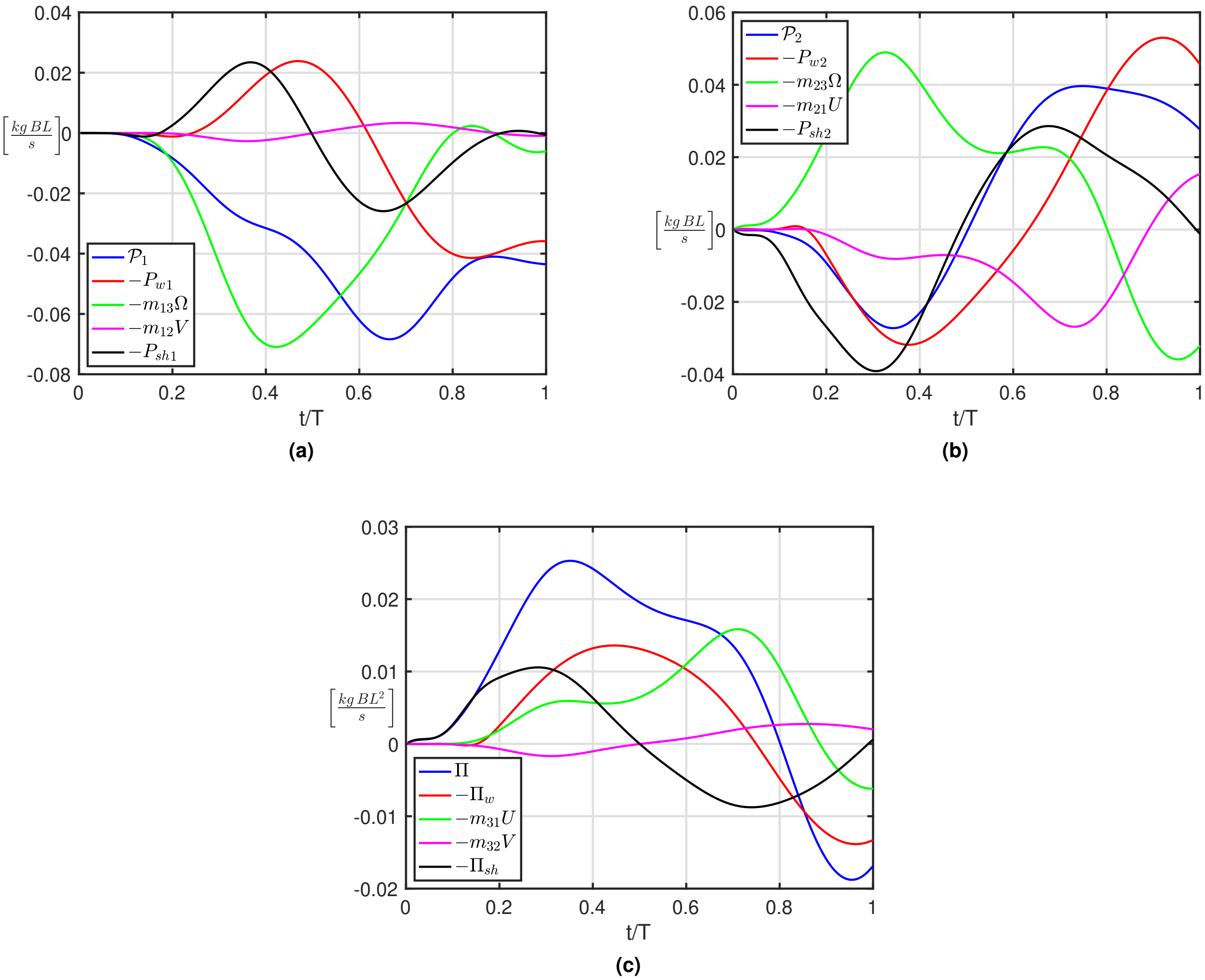

Figure S5. Time history of the impulses contributions for the C-start maneuver in presence of a traveling wave: (a) forward, (b) lateral and (c) angular direction. The curves are very similar to the ones in the absence of a traveling wave despite a certain increase in their absolute value may be appreciated.

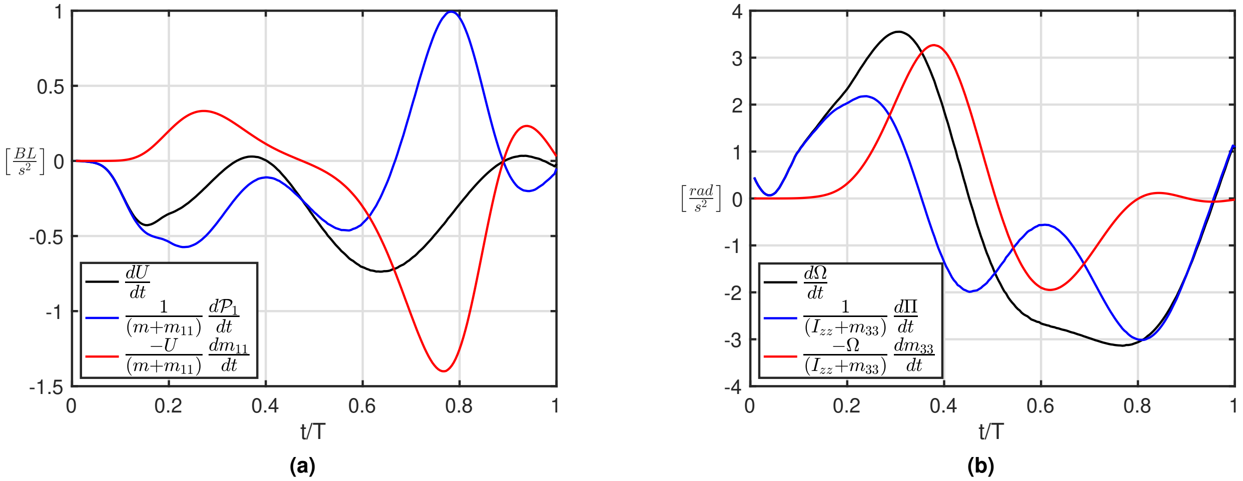

Figure S6. Time history of (a) the forward and (b) the angular acceleration contributions. The curves are very similar to the ones in the absence of a traveling wave despite a certain increase in their absolute value may be appreciated.

Supplementary Video Legends

Movie S1 Animation of the C-start maneuver for a neutrally buoyant fish from the numerical simulation.

Movie S2 Animation of the C-start maneuver with the addition of a traveling wave for a neutrally buoyant fish from the numerical simulation.

References

1. Jayne, B. & Lauder, G. Red and white muscle activity and kinematics of the escape response of bluegill sunfish during swimming. *J. Comp. Physiol. A* **173**, 495–508 (1993).

2. Wakeling, J. M. & Johnston, I. A. Muscle power output limits fast-start performance in fish. *J. Exp. Biol.* **201**, 1505–1526 (1998).

3. Wakeling, J. M. & Johnston, I. A. Body bending during fast-starts in fish can be explained in terms of muscle torque and hydrodynamic resistance. *J. Exp. Biol.* **202**, 675–682 (1999).

4. Liu, G., Yu, Y. L. & Tong, B. G. Flow control by means of a traveling curvature wave in fishlike escape responses. *Phys. Rev. E* **84**, 056312 (2011).

5. Paniccia, D., Graziani, G., Lugni, C. & Piva, R. On the role of added mass and vorticity release for self propelled aquatic locomotion. *J. Fluid Mech.* **918**, A45, DOI: 10.1017/jfm.2021.375 (2021).
